# Implications of Extreme Life Span in Clonal Organisms: Millenary Clones in Meadows of the Threatened Seagrass *Posidonia oceanica*

**DOI:** 10.1371/journal.pone.0030454

**Published:** 2012-02-01

**Authors:** Sophie Arnaud-Haond, Carlos M. Duarte, Elena Diaz-Almela, Núria Marbà, Tomas Sintes, Ester A. Serrão

**Affiliations:** 1 Ifremer, DEEP-Centre de Brest , Plouzané, France; 2 CCMAR, Laboratório BEE, Universidade do Algarve, Faro, Portugal; 3 Department of Global Change Research, IMEDEA (CSIC-UIB), Mallorca, Spain; 4 Oceans Institute, University of Western Australia, Australia; 5 Instituto de Física Interdisciplinar y Sistemas Complejos, IFISC (CSIC-UIB), Mallorca, Spain; University Copenhagen, Denmark

## Abstract

The maximum size and age that clonal organisms can reach remains poorly known, although we do know that the largest natural clones can extend over hundreds or thousands of metres and potentially live for centuries. We made a review of findings to date, which reveal that the maximum clone age and size estimates reported in the literature are typically limited by the scale of sampling, and may grossly underestimate the maximum age and size of clonal organisms. A case study presented here shows the occurrence of clones of slow-growing marine angiosperm *Posidonia oceanica* at spatial scales ranging from metres to hundreds of kilometres, using microsatellites on 1544 sampling units from a total of 40 locations across the Mediterranean Sea. This analysis revealed the presence, with a prevalence of 3.5 to 8.9%, of very large clones spreading over one to several (up to 15) kilometres at the different locations. Using estimates from field studies and models of the clonal growth of *P. oceanica*, we estimated these large clones to be hundreds to thousands of years old, suggesting the evolution of general purpose genotypes with large phenotypic plasticity in this species. These results, obtained combining genetics, demography and model-based calculations, question present knowledge and understanding of the spreading capacity and life span of plant clones. These findings call for further research on these life history traits associated with clonality, considering their possible ecological and evolutionary implications.

## Introduction

Clonal organisms are present in all living kingdoms and play key roles in the biosphere, including making a significant contribution to global primary production [1], [2]. Despite their importance, however, our current understanding of their ecology and evolution is limited by the constraints that the clonal life history trait puts on the use of classical ecological and evolutionary approaches. Such methods rely on the assumption that individuals have different genotypes and limited generation times, whereas these partially asexual organisms may have achieved an alternative optimal evolutionary strategy. Clonal organisms can potentially recombine genetically as much as necessary to avoid the accumulation of deleterious mutations and escape parasites, while being able to maintain the best performing genotypes [3] over time scales far exceeding sexual generation times. In clonal organisms, the maximum life span of a particular genotype is a crucial component of the ‘generation time’ (i.e., the average interval between the birth of a genetic individual and the birth of its sexually generated offspring) and, therefore, influences all evolutionary processes affected by this life history trait. Indeed, the generation time, maximum life span and size reached by clones have considerable implications for the action range of drift, and the spatial and temporal variation of selection regimes.

**Figure 1:**
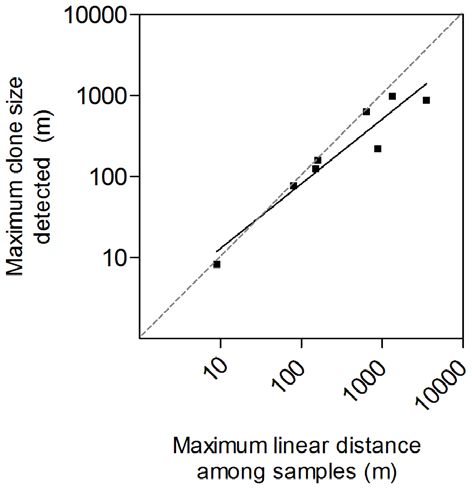
Relationship between maximum clonal size detected and distance sampled in studies reporting very large clones. The solid line shows the fitted regression line of clonal size (S, m) over distance (D, m): log10 S = −0.14 (±0.10)+1.15 (±0.26) log10 D (R2 = 0.91, N = 8, p<0.001). The regression intercept and slope do not differ significantly from 0 and 1, respectively (t-test, p>0.05), thus not rejecting the hypothesis that there is a general tendency for the maximum clonal size detected to be limited by the maximum distance sampled.

Only a few studies have reported estimates of these parameters for clonal organisms, and the largest known clones identified so far, which are fungi [4] and angiosperms [5], [6], [7], extend over hundreds of metres in space and potentially centuries to millennia in time. Additionally, close examination of the sampling strategies used in deriving these estimates strongly suggest that past estimates of the maximum size (and life span when related, see also [8]) of clonal organisms have been limited by the choice of sampling scale (Table 1, Fig. 1). Sampling strategy can be chosen for different research questions and species, but is typically limited to several hundred metres [4], [6], [9], [10], [11], [12], [13], [14], [15], [16], [17], [18], [19], [20], [21], [22], [23], [24]. A strong relationship can be seen between the maximum distance sampled and the maximum estimated clonal size among these studies (Table 1, Fig. 1), suggesting that greater estimates of maximum clonal size could have been derived had greater sampling scales been used. About half of the published reports of very large clones indeed show maximum clonal sizes corresponding to the size of the sampling area (Table 1). Moreover, a good number of studies on clonal organisms do not address the possibility that common genotypes occur across distant localities, suggesting that, despite their important ecological and evolutionary implications, the true maximum size and life span of clonal organisms are often overlooked.

**Table 1:** Maximum size and age reported in the literature for clonal organisms.

Clone identification*	Methods for age estimates†	Species (reference )	Clonal size(m or m^2^)	Age(yr^−1^)	Sampling scale‡(m)
E	CGR & ^14^C	*Larrea tridentata * [*23*]	7.8 m	>11,000	-
-	-	*Lycopodium complanatum * [*10]*, [65**] #	250 m	850	patch
E	CGR & ^14^C	*Picea mariana * [*17*]	14 m	>330	patch
P	CGR	*Festuca rubra * [*12*]	220 m	>1,000	880
P	CGR	*Festuca ovina * [*13*]	8.25 m	>1,000	9
P	-	*Holcus mollis * [*14*] #	880 m	>1,000	3500
PI	CGR	*Calamagrostis epigejos * [*19*] #	50 m	>400	patch
PI	CGR	*Convallaria majalis * [*19*] #	83 m	>670	patch
PI	-	*Populus tremuloides * [*15*] #	507 m	>10,000	patch
-	-	*Gaylussaccia brachycerium * [*24*]	1980 m	>13,000	-
-	-	*Pteridium aquilinum * [*65*]	482 m	1,400	patch
MLG (allozymes)	CGR	*Pteridium aquilinum * [*21*]	390 m	930	patch
MLG (allozymes)	CGR	*Pteridium aquilinum * [*20*]	1000 m	1,180	1328
MLG (allozymes)	^14^C	*Lomatia tasmanica * [*5*]	1200 m	43,600	patch
MLG (RAPD)	CGR	*Vaccinium macrocarpon * [*22*]	125 m	>350	150
MLG (RAPD)	^14^C	*Picea mariana * [*16*]	691 m^2^	>1,800	-
MLG (RAPD)		*Armillaria bulbosa (fungi)* [*4*]	635 m	>1,500	635
MLG (microsatellites)	CGR	*Zostera marina * [*6*]	160 m	>1,000	160
MLG (microsatellites)	CGR .yr^−1^	*Posidonia oceanica * [*9]*, [11**]	80 m	>600	80

For each species, the methods used to discriminate clones and infer their age, the maximum linear clonal extent are given, as well as the estimated age and the linear scale of sampling are detailed, when available.

*E: excavation; P: phenotype; PI: patch identification; MLG: multilocus genotype (marker used).

† CGR: according to clonal growth rate per year; ^14^C: ^14^C estimates.

# *in* Smith et al, 1992.

‡ patch means the samples encompassed the size of the patches in the field.

Seagrasses are clonal marine angiosperms that can form extensive meadows. They support important marine ecosystems that rank among the most valuable on earth in terms of biodiversity and production, but are experiencing a worldwide decline [25], [26]. Understanding the factors influencing seagrass ecological and evolutionary dynamics is critical for forward planning to prevent their decline. Among seagrass species, clones of the *Zostera marina* and *Cymodocea nodosa* have already been shown to reach large genet sizes (genet: term used in clonal plants to refer to clones) attaining several centuries in age [6], [27]. These two species are relatively fast-growing and have shorter-lived shoots, exhibiting a quicker cycle of growth and death of ramets (term used in clonal plants to refer to shoots), than the Mediterranean endemic *Posidonia oceanica*. Size and age attained are highly dependent on the species studied and the mode of clonal growth. Seagrass clonal growth relies on dichotomous branching and rhizomatic extension through cell division at the rhizome meristems, a growth pattern that leads to a predictable correlation between clonal size and age within species [28] and which is likely to minimize the probability of spread of somatic mutations, in contrast to other clonal growth models such as that of aspen [8].

**Figure 2:**
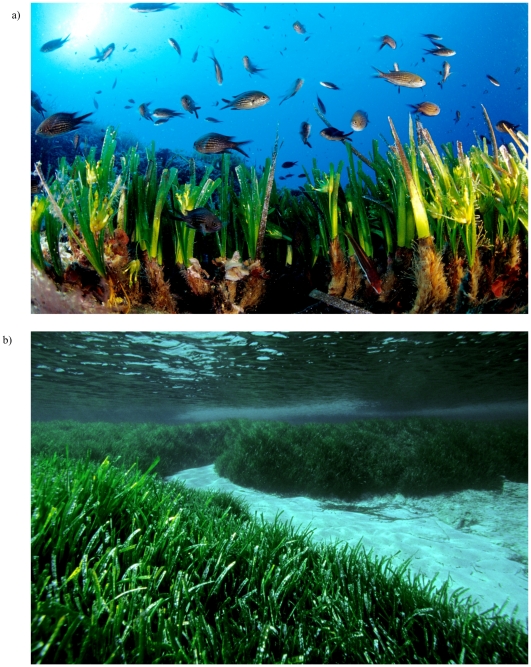
Photograph of meadows of *Posidonia oceanica*. Pictures (a) illustrating the individual shoots (ramets), (b) hosting the largest (15 km) clones detected in this study. Photograph by M. San Félix.

The endemic Mediterranean seagrass *Posidonia oceanica* ranks amongst the slowest-growing and longest-lived plants in existence [29], [30]. This structural species lacks native competitors and major predators in the littoral marine habitat (0–40 m) it occupies, leading to the development of extensive, monospecific meadows ([29], Fig. 2); however, these are presently declining [30], [31] throughout its range. Previous studies provided evidence that *Posidonia oceanica* meadows have grown continuously at particular locations for over 6000 years [32] and that clone mates occur at distances up to at least 80 metres [9], [33] that can only be covered over a minimum of 600 to 700 years of clonal growth (Table 1, see Fig. S1). Altogether these results suggest that *P. oceanica* clones can achieve millenary life spans.

Here, in an attempt to improve estimates of the spatial scales over which identical *P. oceanica* clones spread and, thus, estimate their potential age, we tested whether *P. oceanica* clones were spread across ranges spanning kilometres. We proceeded by deliberately searching for the existence of shared genets among 40 *P. oceanica* populations across the Mediterranean, using microsatellite markers [34], [35], [36] to identify clonal lineages.

## Results

We found 902 distinct multi-locus genotypes (mlg) among the ∼1500 sampling units collected across 40 locations, based on the seven microsatellite markers [34] previously selected for their ability to discriminate among genets from the corresponding mlg [9], [35], [36]. We found no evidence for somatic mutation or scoring errors with the sets of 7 or 9 microsatellites analysed [36]. It was not necessary to define any Multi Locus Lineages (mll, [36]) corresponding to genets including slightly distinct Multi Locus Genotypes that would have diverged through somatic mutation or scoring error, since the statistics supported all different mlg's to be derived from different reproductive events (see below for estimate of somatic mutation rate).

**Figure 3:**
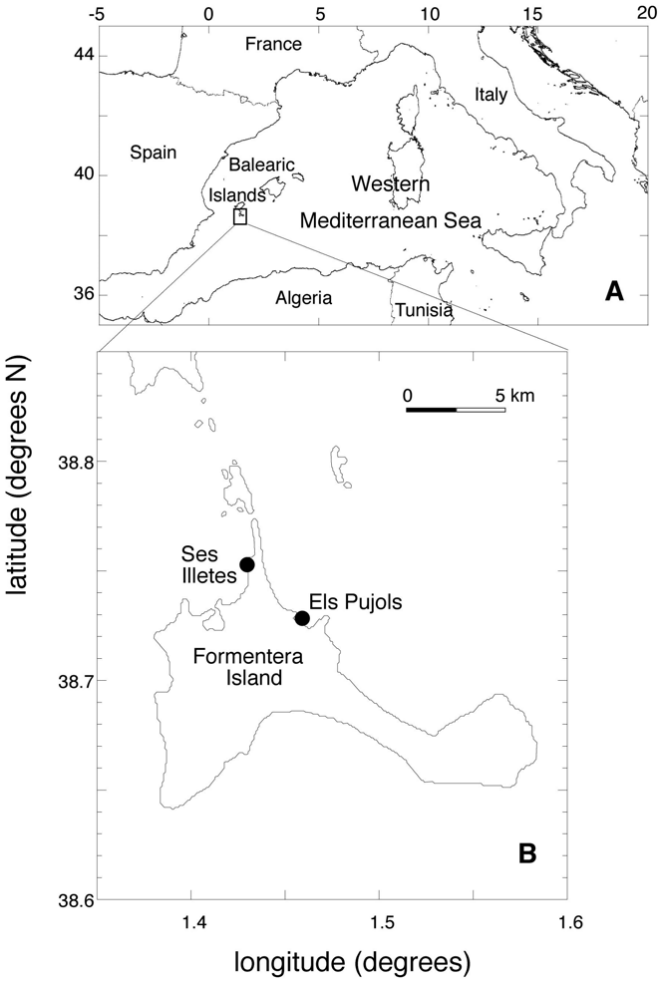
Location of the meadows sampled in Formentera (Balearic Islands, Spain). Map of the location and coastal area of the island of Formentera (A, B) The circles indicate the sites where the shared genotypes were sampled.

Among the 741 pairs of sampling locations, which were separated by up to 3500 km, no shared mlg were found for meadows more than 15 km apart. In contrast, in 5 out of the 21 pairs of locations less than 15 km apart, one to four mlg were shared among distinct locations, sometimes represented by several (up to 10 upon 40) sampling units (shoots, usually referred to as ramets for clonal plants, *see* Fig. 2a and Fig. S1) widespread at each sampling location. In fact, in Formentera Island (Balearic Islands, Spain), up to 18 to 30% of sampled ramets belonged to genets encountered in two localities 15 km apart (Table 2, Fig. 3). In four of the five identified population pairs, the probability that the shared mlg occurred as a result of independent sexual events □ which would have resulted in distinct genets sharing the same mlg □ was very low (p<0.01, and in most cases p<0.001; Table 3). Therefore, the most parsimonious explanation for the presence of identical mlgs in these pairs of locations is that these distant ramets were generated by clonal growth of the same genet. In the fifth case, among sites in Paphos (Cyprus), where p>0.01, the same mlg represented about 25% of each sample. Such dominance lowers the statistical power to ascertain clonal membership, and the non-significance may either be due to the occurrence of different undistinguished genets or to low statistical power.

**Table 2:** Location, demography and clonal characteristics of meadows.

		D	*S* (×10^5^)	N_s_	G	R	*G_tot_*(×10^5^)	% *G*_sampled_	*p_sex(fis)_*	G_s-samples_	% *G*_shared_	*G*_s-quadrates_
Sicily (1 km)	Acqua AzzuraST3	152	2.43	40	31	0.77	1.87	0.017	(10^−5^ to 10^−4^)	2	3.45	20590
	Acqua Azzura ST5	357	5.72	40	29	0.72	4.10	0.007				
*Cyprus (1 km)*	*Amathous ST3*	*484*	*7.74*	*40*	*18*	*0.44*	*3.38*	*0.005*	*0.15^NS^*	*1*	*2.38*	*21135*
	*Amathous ST5*	*559*	*8.94*	*40*	*25*	*0.62*	*5.50*	*0.005*				
Spain (peninsula; 1 km)	El Campello ST3	14	0.23	39	26	0.66	0.15	0.172	10^−2^	2	4.26	2965
	El Campello ST5	60	0.97	40	23	0.56	0.55	0.042				
Spain (Balearic Islands, Formentera; 15 km)	Els Pujols	746	11.93	40	27	0.67	7.96	0.003	(10^−2^ to 10^−6^)	4	8.89	127605
	Ses Illetes	667	10.67	36	22	0.60	6.40	0.003				
Spain (Balearic Islands, Formentera, Ibiza; 7 km)	Sa Torreta	527	0.44	40	21	0.51	4.33	0.005	10^−3^	1	5.00	21633
	Playa Cavallets	-	-	38	28	0.73	-	-				

Details of the geographic locations of the four pairs of populations where shared genets were detected (with the distance between them in kilometres), showing, for each population (except Playa Cavallets for which no demographic data were available), the density of shoots (D; shoots m^−2^), the estimated total number of shoots (S) in the area sampled (1600 m^−2^), the number of shoots sampled in each area (Ns), the number of genets found among these shoots (G), the estimated clonal richness (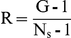), an approximation of the total number of genets in the area sampled (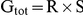) and the expected percentage of this number sampled (% G_sampled_). For each pair of populations, the observed number of genets shared among pairs of samples (Gs-samples), the estimated percentage of genets shared (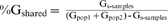), the estimated total number (Gs-quadrats) of genets shared among pairs of meadows present in the areas sampled, and the estimates of the probability that identical genotypes are derived from distinct sexual reproduction events Psex(fis). Results for Cyprus-Paphos- are presented in italics due to the non-significance of Psex(fis) for the mlg shared among localities in this area (see results). No demographic data were available for Playa Cavallets.

**Table 3:** Location and probabilities of shared clones among meadows.

Region	Site	Shared MLG	N_ramets_	*p*_sex_
Sicily	AcquaAzzuraST3&ST5	1	1&1	1.07E-4
		2	1&1	1.7E-5
*Cyprus*	*AmathousST3&ST5*	*1*	*9&10*	*0.15*
Spain	El Campello ST3&ST5	1	1&1	1.96E-2
		2	3&2	9.5E-3
	SesIlletes&Es Pujols	*1*	*5&1*	4.4E-3
		2	10&4	5.3E-3
		3	1&1	9.46E-6
		4	1&2	4.9E-2
	PlayaCavallets&SaTorreta	1	1&1	5.8E-3

Names of pairs of localities sharing identical MLG, identical MLG detected, number of ramets sharing an identical multilocus genotype in each meadow, and probability for the identical MLG to be issued from distinct sexual events. Results in italics correspond to the occurrence of common genotypes for which identity of distinct genets could not be discarded at the alpha = 5% level.

The combination of demographic data [31] with data on clonal diversity [9] for the populations we studied indicates that the number of genets (distinct mlg) expected in each of the sampling areas (the 80 m×20 m area from which shoots were taken randomly) is very large (15,000 to 795,000, Table 2). Bearing this in mind, the fact that we found a large percentage of shared genets in the sample pairs (up to 8.9% of sampled genets, Table 2) despite the small sample size collected at each site (0.003 to 0.1% of the ramets present in the sampling area, Table 2) indicates that shared genotypes must indeed be very common in meadows less than 15 km apart. We estimated that the number of shared genotypes between two sampled areas in Formentera must attain 127,000 to yield the observed percentage of shared mlgs (Table 2), thus supporting the presence of a high prevalence of shared genotypes between these meadows. Although the lower number of shared genotypes in the other pairs of locations renders those estimates subject to higher uncertainties, they also suggest a high rate of shared genets in those meadows, with several thousands to tens of thousands of genets potentially shared between any two meadows less than 15 km apart. Moreover, although no shared genets were detected in the remaining 15 population pairs (70% of all possible pairs) sampled within a spatial scale of 15 km, the number of shared genotypes may still range from 1.5 to 10% of the total number of genotypes present (i.e., up to 80,000 shared genotypes in pairs of sampling areas; Table 2), the limit of detection possible with our sampling effort, and yet remain undetected in our samples.

**Figure 4:**
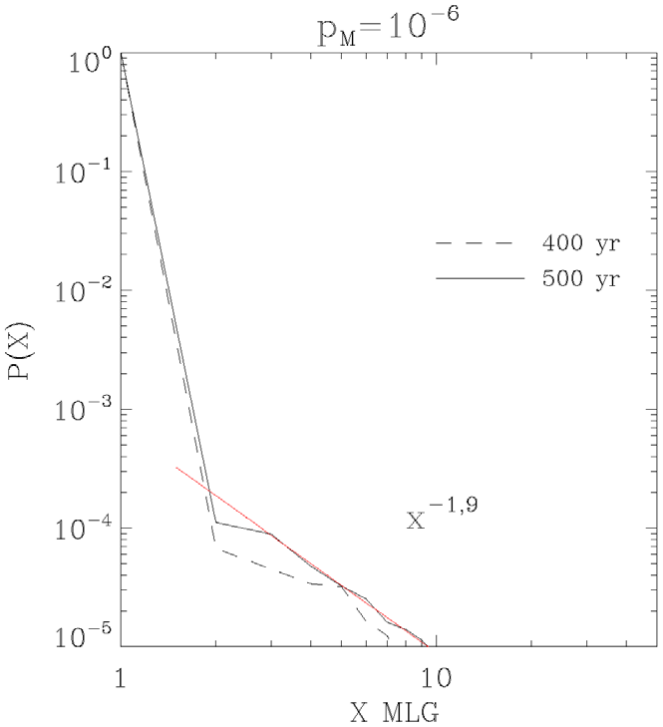
Ranked proportions of the genets (X) belonging to an MLG present in the meadows. from X = 1, the original genet, to X = 2…n representing the genets derived from somatic mutations, ranked by abundance of ramets. The results are derived from somatic mutations in meadows growing over 400 to 500 years with an overall probability (across nine loci) pM = 10-6. The original ramet, X = 1, is the most abundant one, P(X = 1) = 0.99978, whereas the abundance of mutants, P(X>1), follow a power-law decay that strongly depends on the somatic mutation probability.

Under a hypothesis of exclusively clonal spread, the models of clonal growth for *P. oceanica* with an estimate of 4 cm.yr^−1^ lead to estimates of clonal growth time of about 10,000 to several tens of thousands of years for mlg growing across meadows 1 to 15 km apart. Given the slow growth rate of *P. oceanica*, clones that extend over kilometres may be expected to have accumulated somatic mutations, although none were detected. The apparent lack somatic mutation in the sampling does, however, agree with results obtained through modelling performed using a rate of genotypic change lower than *p*_M_ = 10^−5^ (P(mlg_sampled_ = mlg_1_) = 0.99005 for *p*_M_ = 10^−5^ and 0.99978 for *p*_M_ = 10^−6^). Our modelling showed that the exponential nature of seagrass clonal growth leads to a disproportionate numerical dominance of the original mlg, even in the presence of somatic mutation. Such a large numerical advantage of the initial mlg_1_ (Fig. 4) results in a rather low probability of sampling any mutant in our dataset for *p*_M_ = 10^−6^ (P(mlg_sampled_ = mlg_1_)∧400 = 0.1, against 0.98 for *p*_M_ = 10^−5^). The results reported here are, therefore, in agreement with a maximum rate of somatic mutation at microsatellites of about *p*_µs_ = 10^−6^ to 10^−7^ per locus, which is closer to the lower bound estimated for the poplar *Populus tremuloides* [8] than to that of the western redcedar *Thuja plicata* [37].

## Discussion

The finding of clone mates 1 to 15 km apart, with an exceedingly low sampling effort (about 1 in 10,000 to 1 in 100,000 of the shoots sampled in each site) suggests the occurrence of a large number of clones spreading on a scale of kilometres across genetically-differentiated meadows [9], [11]. A clonal spread of *P. oceanica* on such a scale is impressive and calls for an assessment of potential confounding factors. Homoplasy is extremely unlikely to account for completely identical genotypes over 9 loci. Indeed, a biogeographic study of *P. oceanica* across its full distribution range, including distances where homoplasy would be more likely, provided no evidence for its occurrence [9]. In addition, the probability that the spread of genets across km-scale distances can be accounted for by independent sexual events yielding identical mlg is exceedingly low (Table 3). Three alternative scenarios could account for our observations. The km-scale spread of clones may result from i) clonal growth alone; ii) rhizome fragmentation and dispersal of thousands of shoots across these scales; or iii) a combination of both spread through clonal growth across large distances and range extension through edge dispersal of fragments.

The scenario of a km-range spread achieved exclusively through clonal growth requires that the clones reach a minimum age of about 12,500 years. Applying the same estimates to the genets shared between the two pairs of meadows, located 7 km apart between Formentera and Ibiza and 15 km apart around a cape in Formentera (Fig. 3), yields a minimum age estimate between 80,000 and 200,000 years, projecting the origin of the clones well into the late Pleistocene. Although there is no biologically compelling reason to exclude this possibility, we consider it to be an unlikely scenario because local sea level changes during the last ice age (from −80,000 to −10,000 years) would place these sampling locations on land (the sea was 100 metres below its present level). The dominance of identical clones on both sides of the island may, therefore, be explained by the occurrence of even older clones that would have been split during glaciation and spread upwards into the newly inundated areas, tracking sea level rise.

The second scenario involves km-range shoot dispersal. Although successful vegetative dispersal through drifting shoots has not been observed in long term surveys of established meadows, it has been reported to contribute to about 70% of patch recruitment in two colonizing sites over a four-year study [38]. Dispersal and successful reattachment of drifting shoots is a diffusive process, likely to decline exponentially with distance. The spread across kilometre spatial scales via this mechanism is, therefore, expected to be a very rare phenomenon, unlikely to be detected with the limited sampling power of this study. An extrapolation of our data (Table 2) suggests that km-range drifting of shoots should have occurred about 2,000 to 127,000 times for the pairs of meadows sharing clone mates in order to result in the number of events we found (Table 2). The thousands of repeated events of drifting shoots following the same trajectories before becoming successfully settled requires a sequence of improbable events that renders this scenario implausible. This scenario is particularly unlikely when one considers that all pairs of locations sharing genotypes showed a significant level of genetic differentiation that is not compatible with a massive exchange of fragments [9], [11].

A canonical, more parsimonious scenario may include the detachment of shoots at the edge of meadows and their reattachment at relatively short distances, generating a stepping-stone model of spread combining clonal growth with vegetative dispersal. Recent evidence of the mosaic nature of *Zostera marina* meadows [39] suggests that meadows are composed of distinct patches resulting from a history of successive local extinction and recolonization by a small set of propagules or shoots within a narrow window of time following local depletion. Although detachment of the incomparably thicker and stronger rhizome of *P. oceanica* would be considerably less frequent, such events may have punctuated the long-term evolution of meadows and the events of (re)colonization. Large, millenary clones spanning across long distances may thus further extend through the detachment and subsequent reattachment of shoots, accelerating the spread otherwise reached exclusively through clonal growth. We consider this model as the most parsimonious with the knowledge available. It implies a less extreme life span than the exclusive clonal growth model, but still requires long-term clonal growth over thousands years, in both potential source and ‘edge’ locations, for clones to be detected in both samples.

The inference of an extreme age of *P. oceanica* clones is supported by results from a numerical model of clonal growth (described below) examining the spread of somatic mutation within genets and assessing the resulting dynamics of the frequency distribution of genet size over time. First, the model confirms the dominance of large genets as illustrated by a power-law distribution of genet size within the meadow when genets approach 500 years of age (the limit of the model; Fig. 4) conforming to the observations made in 40 meadows of *P. oceanica* examined here). Additionally, the model supports a probability of occurrence of somatic mutation lower than 10^−6^, which would explain the lack of mutants detected in the sampling, and which underlines the persistent numerical advantage of the original mlg over slightly distinct ones emerging through somatic mutations.

Finally, these results are consistent with the phylogeny of the *Posidonia* genus obtained through sequencing of distinct genes from the mitochondrial, chloroplastic and nuclear genomes [40]. The complete lack of polymorphism within *P. oceanica* across its whole distribution range at all tested genes including ITS, and its very low divergence from its Australian counterparts, lead to an estimate of evolutionary rates among the slowest reported for herbaceous plants. The highly conserved genetic structure of the *Posidonia* genus suggests low mutation rates and long generation time of genets [41], [42] as a likely explanation for such low genetic divergence.

In this study, we found evidence supporting the occurrence of extremely large clones (up to 15 km) and life spans of thousands to tens of thousands of years. These *P. oceanica* genets would have coped with environmental variation over long periods of time and are presently distributed in microhabitats kilometres apart. They would, therefore, be expected to display large phenotypic plasticity. Reaching such an old age has been suggested to have at least two major evolutionary implications for a clonal lineage [43], [44]. One is the lack or limited accumulation of deleterious mutations that would otherwise eventually lead to extinction: the so called “Muller's ratchet”, which is predicted under certain population parameters. Our results suggest that this accumulation might be limited or hampered due to large population size [45], as well as by the persistent dominance of the original genotype under a pure regime of clonal growth demonstrated by the present model (Fig. 4). The other is a potentially high phenotypic plasticity providing long-lived individuals with the capacity to survive in a changing environment.

Two evolutionary models have been proposed to explain the ubiquity of clonal organisms: the General-Purpose Genotype model [46] and the Frozen Niche Variation model [47]. The first explains the ubiquity of clonal organisms by the ability to retain the most competent genotypes over time (i.e., “*the gene complexes most co-adapted* to the prevailing conditions [48]”), when “*a single event of outcrossing may destroy such genic assoc*i*ation* [48]”. The second model explains the co-occurrence of diverse sexual and asexual genotypes by their adaptation to narrow, non-overlapping, specialist niches. Both theories have been supported by empirical observations on different organisms. The results reported here suggest that there is phenotypic plasticity in particular genotypes, allowing adaptation to microhabitats spreading over kilometres and to fluctuating mid or long term environmental conditions; this would support positive selection of “general purpose genotypes” [46]. Such a mechanism would, in turn, favour the absence of recombination and, therefore, of sexual reproduction, once an optimal genotype was reached. This is supported by direct observations of very infrequent sexual reproduction in *P. oceanica* [38], [49], suggesting that molecular estimates of the proportion of sexually derived recruitment [9], [35] are likely to correspond to the integration of rare successful sexual episodes during narrow windows of time rather than frequent or present day sexual output, as already reported by Balestri and Lardicci [50]. Although genet diversity has been shown to enhance short term response and ecosystem resistance of *Zostera marina* in experimental plots [51], [52], low genet diversity accompanying large clone size coincides with improved capacity to respond to disturbances, such as an increased organic load imposed by fish farming on natural *P. oceanica* meadows [53], [54]. This further supports the hypothesis of phenotypic plasticity associated with large clonal size and old age. Nevertheless, even though such phenotypic plasticity possibly evolved across millennia, it may well be challenged by the unprecedented rate of environmental change imposed by current global climate change [55], including temperature increase and ocean acidification, and recent anthropogenic pressure on coastal areas resulting in changes in water quality, eutrophication, and nutrient load, particularly in seagrass meadows [56].

### Conclusions

The finding of *P. oceanica* clones (i.e., genets) that are extreme in size (km-sized) and age (multi-millenary old) across the Mediterranean indicates that some meadows are the result of ecological and evolutionary processes integrated over long time scales. Time scales such as these are in a stark contrast to the current rapid and acute impact caused directly or indirectly by human pressure on this species. Indeed, the ancient meadows of *P. oceanica* are declining at a rate several hundred-fold faster (about 5%.yr^−1^, [31], [55]) than the rate over which they spread when forming [28], [57], a situation that this slow growing, long-lived species is poorly capable of recovering from.

Critical screening of the literature and the results presented here suggest that a systematic search for temporal persistence of clones has not yet been attempted for most species, and that extreme potential to persist over large spatial and temporal scales may be a common feature associated with clonality. This finding, if confirmed by further specific efforts to test for large clonal size, would have important implications for ecological and evolutionary theory, as all basic parameters in evolutionary ecology. Effective population size and consequent drift, migration, mutation and selection would be strongly influenced by the potentially extreme generation overlap caused by life spans reaching thousands to possibly tens of thousands of years. Our results underline the need to improve our knowledge of the life span of clonal organisms, as some assumptions underlying classical population models may not be met by taxa with ancient and large clonal lineages, such as the Mediterranean seagrass *Posidonia oceanica*.

## Materials and Methods

### Sampling

*P. oceanica* samples were taken at total of 40 sites across the Mediterranean basin from Spain to Cyprus by collecting about 40 shoots at randomly selected coordinates within a 1600 m^2^ (80×20 m) area at each site [9], [11]. The basal meristematic section of the leaves was removed and preserved in silica crystals until analysis. Twenty six of these meadows were analysed in previous studies on biogeography [9] and the effects of aquaculture installations on *P. oceanica* meadows [11]; the other 14 were added in the present study (Table S1). Sampling locations were separated by approximately 1 to 3500 km.

All 40 sampling quadrats were located in non-privately-owned or specifically protected areas, so no specific permits were required for the field studies. *Posidonia oceanica* is not considered as an endangered (its status is ‘least concern’ in the IUCN list) or protected species, although the meadows it constitutes are a protected habitat.

### Genotyping

Genomic DNA was isolated following a standard CTAB extraction procedure [58]. Each shoot or group of connected shoots sampled together were treated as a “sampling unit” (SU), or ramet. All meadows were analysed with the most efficient combination [35] of 7 dinucleotide nuclear markers [34], Po4-3, Po5, Po5-10, Po5-39, Po5-40, Po5-49 and Po 15, as described in [9]. When identical genotypes were found across sites with a probability higher than 1% of being issued from distinct sexual reproduction events rather than from clonal growth (see here-below), two additional loci, Po4-36 and Po5-61 [34], were genotyped in order to ascertain genet membership with higher confidence.

### Methods used for clone (genet) discrimination

When the same genotype was detected more than once, the probability that the samples actually originated from distinct reproductive events (i.e., from separate genets) was estimated [20], [59] using the software GenClone [60], taking into account Wright's inbreeding coefficient estimated for each locus [60].

The possible occurrence of somatic mutations or scoring errors leading to slightly distinct mlg actually derived from a single reproductive event, i.e., from one single clone, was also tested for. This screening aimed to identify, if they occurred, Multi Locus Lineages (mll), including slightly distinct mlg originated from the same zygote, as mentioned above [9], [36].

### Genotypic richness

Genotypic richness was estimated from the number of ramets sampled (N) and the number of multilocus genotypes detected (G), as suggested by Dorken *et al.* [61]:
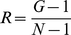
(3)


### Demographic estimates

In each meadow studied for demography, the number of shoots was counted within three replicated quadrats, varying between 0.09 m^2^ and 1 m^2^ in area depending on the sites (so as to include at least 100 shoots quadrat^−1^). The only exception was one station influenced by the presence of aquaculture cages (El Campello, Spain), where the quadrat was 10 m^2^ [11]. More details on the demographic estimates are available in Marbá et al. [31].

### Estimates of shared genets and type II errors: likelihood of undetected existence of shared genets

An approximation of the total number of genets in the area sampled was obtained as follows, with S the estimated number of shoots in the area sampled:
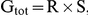
allowing an estimate of the expected percentage of these sampled % G_sampled_ as:
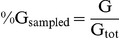
For each pair of populations, the observed number of genets shared among pairs of samples (Gs-samples) was then used to estimate the total percentage of genets shared :
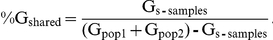
The likelihood of occurrence of undetected shared genets among all pairs of localities separated by less than 15 kilometres was estimated. For each meadow pair, an estimate was made of the maximum possible percentage of shared genets, by considering that the addition of one single genet to one of the samples may allow the observation of a new, shared, previously undetected genet. The estimate of the maximum percentage of shared genets between the meadows that may still remain undetected in our sampling was, therefore, considered for each pair of meadows i and j with G_i_ and G_j_ genets:
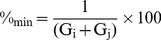
(4)


### Modelling of clonal growth and spread of somatic mutations

We modified an existing model of *P. oceanica* clonal growth [28], [62], [63] to derive quantitative expectations of the number of somatic mutations we would expect to be detected and to compare these estimates with the data available on the genetic structure of meadows. More specifically, the model was modified to introduce the occurrence of random somatic mutations and to study their quantitative spread within a genet across extended time spans (500 years). The model is initiated with a seed, growing to occupy space through clonal growth. Each new shoot has a probability *p*_M_ to modify its genotype with respect to its neighbour along the rhizome. The model tracks the fate of all individual shoots and rhizome meristems produced during the clone's development to yield a spatially-explicit representation of the clone at various time steps [63]. At the same time, the model output contains descriptors of the changes over time in the average number of surviving shoots, internal shoot density, patch radial expansion and growth rate, as well as the distribution probability and abundance of the different genotypes arising from somatic mutations during clonal growth. In order to derive a conservative estimate of age through clonal growth modelling, the highest bound estimate of spatial clonal growth rate estimated for *P. oceanica* was used (6 cm.yr-1), and the maximum distance was divided by two, assuming a starting point for clonal spread in the midpoint between the two most distant ramets sharing the same mlg in the sampling.

The expected mutation rate at microsatellites for mitotic divisions may be ten to hundred fold lower than the mutation rate per sexual generation in higher eukaryotes [64]. Estimates of the rate of somatic mutations are still scarce overall, highly variable between different organisms and still mostly only available for trees, with values ranging from 4.10^−3^ to 6. 10^−4^ in the western redcedar *Thuja plicata* [37] and 4.10^−5^ and 6.10^−7^ for the poplar *Populus tremuloides* [8]. We explored a range of three orders of magnitude in somatic mutation with overall probability of genotypic change *p*_M_ = 5. 10^−3^, 10^−4^, 10^−5^ and 10^−6^, corresponding to a rate of somatic mutation of about *μ*_s_ = 1.5×10^−3^ to 10^−7^ per locus for the seven to nine loci used to discriminate identical mlg. The amount of ramets for the original multi locus genotype (mlg_1_) compared to ones that were slightly different due to somatic mutations (mlg_x_) were estimated for all rates in patches that had been growing for 100 to 500 years (p_1_). The most dominant genets detected in real samples of 40 shoots were sampled between 5 and 10 times and no somatic mutations were detected with a set of seven to nine loci. The probability of sampling only the initial mlg_1_ in all 40 meadows where each dominant mlg was on average sampled 10 times was roughly estimated as p_1_∧400. The ranked abundances of mlg_1_ and the mlg derived from this initial one through somatic mutations were then built in order to illustrate the dominance of mlg_1_ (from which the sampling probability p_1_ is derived) depending on the probability of genotypic change *p*_M_ used to run the model. Finally, p_1_∧400 was estimated in order to test whether the *p*_M_ and corresponding somatic mutation rate *μ*_s_ were compatible with the lack of observation of somatic mutation in the set of samples analysed in this work, which contained about 1500 sampling units analysed in this work.

## Supporting Information

Figure S1 Mapping of clones extent and distribution. Multiple occurrence of large genets mapped in adjacent meadows is detailed in (a) and (b) Amathous ST3 and ST5 (for which shared genet is labelled as 4 and 19, respectively), (c) Es Castell (where the largest clone was encountered), and (d) Los Genoveces.
(DOC)

Table S1 Sampling details. Sampling regions and localities, approximate GPS coordinates, approximate depth in meters (D), number of sampling units analyzed (N_s_).
(DOC)
