# Supplementary material for: Implications of Extreme Life Span in Clonal Organisms: Millenary Clones in Meadows of the Threatened Seagrass Posidonia oceanica
Source: PLoS One. 2012 Feb 1;7(2):e30454. doi: 10.1371/journal.pone.0030454 (PMC3270012; doi:10.1371/journal.pone.0030454)
Supplement: Figure S1 — Mapping of clones extent and distribution. Multiple occurrence of large genets mapped in adjacent meadows is detailed in (a) and (b) Amathous ST3 and ST5 (for which shared genet is labelled as 4 and 19, respectively), (c) Es Castell (where the largest clone was encountered), and (d) Los Genoveces. (DOC) [file pone.0030454.s001.doc]

**Figure S1**

a)

b)

c)

d)
