# Supplementary material for: Implications of Extreme Life Span in Clonal Organisms: Millenary Clones in Meadows of the Threatened Seagrass Posidonia oceanica
Source: PLoS One. 2012 Feb 1;7(2):e30454. doi: 10.1371/journal.pone.0030454 (PMC3270012; doi:10.1371/journal.pone.0030454)
Supplement: Table S1 — Sampling details. Sampling regions and localities, approximate GPS coordinates, approximate depth in meters (D), number of sampling units analyzed (Ns). (DOC) [file pone.0030454.s002.doc]

**Supporting Information**

**Table S1**

|  | Sampling localities | GPS | coordinates | D | N_s_ |
| --- | --- | --- | --- | --- | --- |
| *Spain (peninsula)* | Roquetas | 2°37.09W | 36°43.26'N | 4 | 50 |
|  | Los Genoveces | 2°07.02W | 36°44.40'N | 8 | 40 |
|  | Rodalquilar | 2º00.53W | 36º51.21'N | 4 | 40 |
|  | Calabardina | - | - | 4.5 | 40 |
|  | Carboneras | 1º53.20W | 36º59.51'N | 4 | 40 |
|  | El Campello ST3 | 0º0.57'E | 38º25.25'N | 28 | 40 |
|  | El Campello ST5 | 0º20.86'E | 38º24.88'N | 27.5 | 40 |
|  | La Fossa Calpe | 0°4.56'E | 38°33.59'N | 3 | 40 |
|  | Campomanes | 0º20.94'E | 38º37.54'N | 7 | 31 |
|  | El Arenal-Calpe | 0º3.06'E | 38º38.37'N | 6 | 39 |
|  | Las Rotes | 0º8.56'E | 38º50.03'N | 6 | 40 |
|  | Xilxes | 0º8.07'E | 39º45.13'N | 13 | 32 |
|  | Torre de la Sal | 0°10.72'E | 40°08.13'N | 9 | 40 |
|  | Punta de Fanals | 2º50.56'E | 41º41.58'N | 17 | 38 |
|  | Cala Giverola | 2º57.44'E | 41º44.13'N | 10 | 40 |
|  | Cala Jonquet | 3º17.36'E | 42º18.19'N | 6 | 39 |
|  | Port lligat | 3º17.58'E | 42º17.61'N | 8 | 40 |
| *Spain (Balearic islands)* | Es Caló des Oli (Formentera) | 1º24.16'E | 38º43.49'N | 5 | 40 |
|  | Illetas (Formentera) | 1º25.83'E | 38º45.37'N | 7 | 35 |
|  | Es Pujols (Formentera) | 1º27.27'E | 38º43.74'N | 5 | 40 |
|  | Sa Torreta (Formentera) | 1°25.18'E | 38°47.45'N | 9 | 40 |
|  | Playa Cavallets (Ibiza) | 1°24.25'E | 38°50.99'N | 4 | 40 |
|  | Porto Colom (Mallorca) | 3º16.18'E | 39º25.05'N | 6 | 50 |
|  | Magaluff (Mallorca) | 2°32.60'E | 39°30.25'N | 4 | 40 |
|  | Es Castell (Cabrera) | 2º55.83'E | 39º9.16'N | 5 | 40 |
|  | Sta. María 13m (Cabrera) | 2º56.92'E | 39º9.07'N | 13 | 35 |
|  | Sta. María 7m (Cabrera) | 2º56.96'E | 39º9.00'N | 7 | 40 |
|  | Addaia (Menorca) | 4º12.42'E | 40º0.97'N | 8 | 40 |
|  | Fornells (Menorca) | 4º08.26'E | 40º3.39'N | 10 | 40 |
| *Italy (Sicily)* | Marzamemi | 15º00.49'E | 36º43.29'N | 8 | 38 |
|  | Acqua Azzura ST3-4 | 15º08.44'E | 36º42.71'N | 22 | 40 |
|  | Acqua Azzura ST5 | 15º08.48'E | 36º43.31'N | 20 | 40 |
| *Tunisia* | Tunis | 10°19.00'E | 36°46.00'N | <10 | 40 |
| *Malta* | Malta 1 | 14°33.00'E | 35°51.00'N | 5-20 | 39 |
| *Greece* | Agios Nicolaos | 23º55.62'E | 37º42.97'N | 6 | 40 |
|  | Anavyssos-Sounion ST3-4 | 23º57.29'E | 37º39.59'N | 16 | 40 |
|  | Anavyssos-Sounion ST5 | 23º58.24'E | 37º39.55'N | 16 | 40 |
| *Cyprus* | Paphos | 32°26.23'E | 34°43.54'N | 10 | 38 |
|  | Amathous ST3 | 32°12.00'E | 34°41.96'N | 20,5 | 40 |
|  | Amathous ST5 | 33°12.98'E | 34°42.02'N | 19,5 | 40 |
